# The peritubular reinforcement effect of porous dentine microstructure

**DOI:** 10.1371/journal.pone.0183982

**Published:** 2017-08-31

**Authors:** Rong Wang, Lin Niu, Qun Li, Qida Liu, Hong Zuo

**Affiliations:** 1 State Key Laboratory for Strength and Vibration of Mechanical Structures, School of Aerospace, Xi'an Jiaotong University, Xi'an, P.R. China; 2 Clinical Reaserch Center of Shaanxi Province for Dental and Maxillofacial Diseases, College Stomatology Hospital of Xi’an Jiaotong University, Xi'an, P.R. China; Georgia Regents University, UNITED STATES

## Abstract

In the current study, we evaluate the equivalent stiffness of peritubular reinforcement effect (PRE) of porous dentine optimized by the thickness of peritubular dentine (PTD). Few studies to date have evaluated or quantitated the effect of PRE on composite dentine. The miscrostructure of porous dentine is captured by scanning electron microscope images, and then finite element modeling is used to quantitate the deformation and stiffness of the porous dentine structure. By optimizing the radius of PTD and dentine tubule (DT), the proposed FE model is able to demonstrate the effect of peritubular reinforcement on porous dentine stiffness. It is concluded that the dentinal equivalent stiffness is reduced and degraded with the increase of the radius of DT (i.e., porosity) in the certain ratio value of *E*_*p*_/*E*_*i*_ and certain radius of PTD, where *E*_*p*_ is the PTD modulus and *E*_*i*_ is the intertubular dentine modulus. So in order to ensure the whole dentinal equivalent stiffness is not loss, the porosity should get some value while the *E*_*p*_/*E*_*i*_ is certain. Thus, PTD prevents the stress concentration around DTs and reduces the risk of DTs failure. Mechanically, the overall role of PTD appears to enhance the stiffness of the dentine composite structure. These results provide some new and significant insights into the biological evolution of the optimal design for the porous dentine microstructure. These findings on the biological microstructure design of dentine materials are applicable to other engineering structural designs aimed at increasing the overall structural strength.

## Introduction

Dentine, as measured by either weight or volume, is the major component in individual teeth and exhibits a complex hierarchical structure with both organic and inorganic components [1]. Assement of the mechanical properties of dentine, such as strength, fatigue, mastication, caries, and abrasion, provide us with a better understanding how dentine responds to its environment. Mature dentine is composed of approximately 70% mineral (i.e. carbonated apatite), 20% organic materials (primarily type I collagen), and 10% water by weight [2]. One of the most distinct microstructural features in dentine is the tubular network that extend outward from the pulp towards the dentine—enamel junction. Each tubular lumen is surrounded by a peritubular and a highly mineralized zone, which consists of a hyper-mineralized collagen-poor region (approx. 0.5 to 1 μm in thickness) of apatite crystals, called peritubular dentine (PTD) [1, 3, 4]. Intertubular dentine (ITD) occupies the region between the tubules and consists of an organic matrix (collagen fibrils) reinforced by nanoscopic apatite crystals similar to that of peritubular dentine [5–7]. The PTD is irregular and not uniformly distributed around the dentine tubules (DTs). The texture of the PTD mineral is different than that of the ITD mineral, and it is difficult to suggest any function of PTD, besides it could provides for the transport of ions and other components [8]. Around tubules, the collagen fibrils are randomly distributed in intertubular dentine. It’s the highlighted microstructural characteristics in dentine. There are different compositions between the peritubular and intertubular dentine and the main components of dentine as an anisotropic composite materials, which determines that the dentine is deemed as a biological composite. And the dentine tubules are also concerned as the particular characteristic [9, 10].

Since dentine is the irregular and porous material microstructure, it leads that the micromechanical modeling of porous dentine is complicated. Dentine also can be considered as a composite material structure. It mainly contains three hierarchical phases such as intertubular inclusion, bonding matrix, and water. In the analysis of dentine configuration, the dentine is regarded as two-phase composite material. One is the dentine tubules which are considered as defects in composite material. The other is the peritubular dentine which is considered as the reinforcement phase, and it is used to compensate the losing stiffness.

Our studies show that the peritubular dentine around DTs has interesting effects on the stiffness of composite dentine structure. Biologically, the porosity associates with the presence of the DTs can serve as a path for neural conduction. However, mechanically, the potential impact of this porosity is still unclear. In addition, the attribution for the complexity and great thickness of the PTD microstructure also remains unclear. Therefore, the main prurpose of this study is to further evaluate the mechanical and structural properties of PTD to gain insight into its biological function.

## Materials and methods

### Ethics statement

All the experimental procedures involving human tooth were approved by the Medical Ethics Committee of Stomatology Hospital of College of Medicine, Xi'an Jiaotong University. All patients provided written informed consent.

### Experiment

The compression experiment test was performed using *in-situ* scanning electron microscope (SEM). The SEM images were obtained which provided the modeling data and served for the following finite element model. The micro-structural evolution were recorded under different compression loading to verify the finite element analysis results about the PTD function. After compression loading, the compression stiffness also could been calculated by each load-displacement curve. The compression stiffness is considered as the verification results to finite element analysis results.

**Fig 1:**
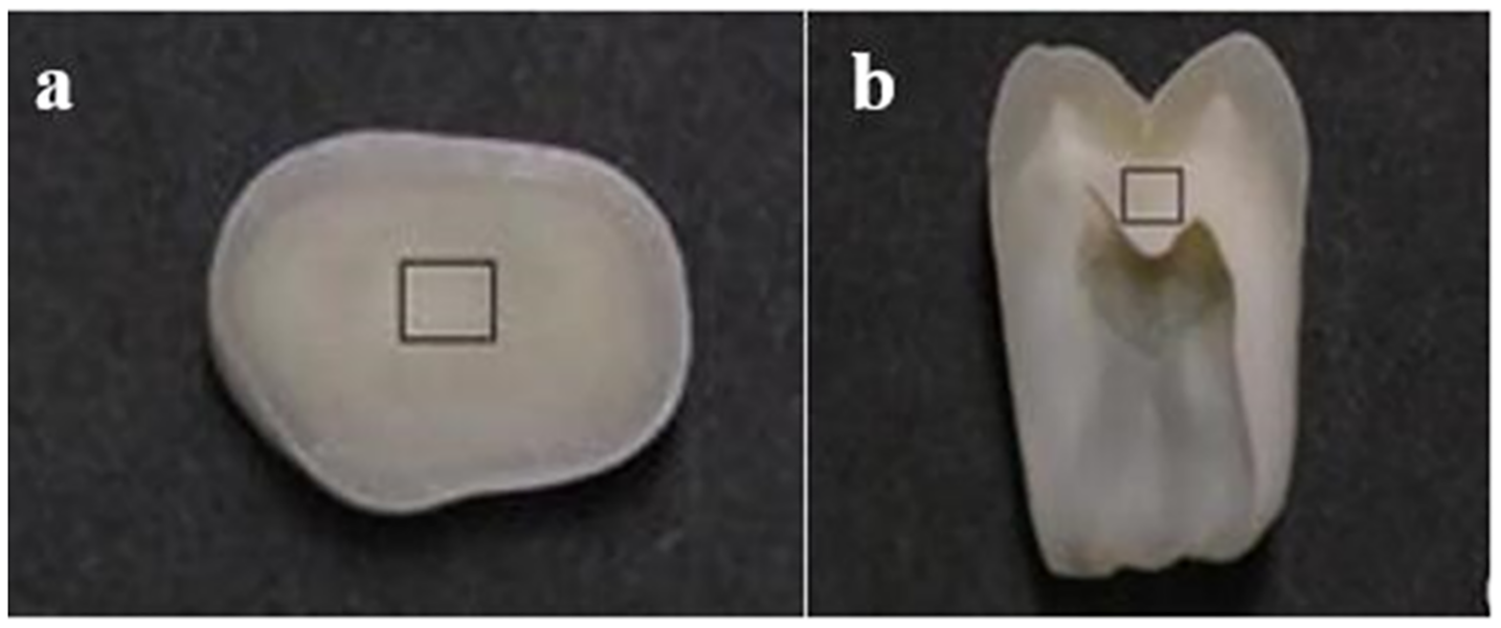
The sectioned and cut samples from the teeth. (a) the cross section of dentine; (b) the cross section of DTs’ direction diagram.

Five non-carious third molars were used for testing, the samples were discard material and thus exempt from the need for Institutional Review Board approval. Teeth were extracted from healthy male and female patients between the ages of 20–40. Because the third posterior molars had to suffer the bite force in general. It had higher requirement in strength property which had more suitable for our study. After extraction, the teeth were kept in normal saline solution at 4°C°C. Before cutting, the tooth was embedded in epoxy resin. The long axis of the teeth was parallel to the mold, and the collisional surface was exposed. The samples were sectioned by a low-speed precision cutting machine (ISOMET1000, Bucheler, USA) with water cooling. The five samples were sectioned at a specific location in the teeth with cubic dimensions of 1.5 mm^3^ as shown in Fig 1, which were used for testing.

Each sample surface was polished in multiple steps using an automatic polishing machine (automat 250, Bucheler, USA). The samples were polished with a series of SiC abrasive papers down to 4000 mesh and finally with 9 μm, 3 μm, and 1 μm diamond suspensions (automat 250, Bucheler, USA). The surface morphology was assessed (Keyence Japan) and the zone in which the tubules were arranged perpendicular to the surface was chosen as the compression surface contacted with the cross-header of the machine.

The MTEST-5000-situ loading machine (SUPRA55, Germany) was equipped with a loading station, displacement sensor, force sensor, propeller lever and software control system. In the loading process, the control system regulated the propeller lever movement, then the displacement and force datas were recorded. Meanwhile, the micro-structure of the sample surface could be observed *in-situ* by SEM.

**Fig 2:**
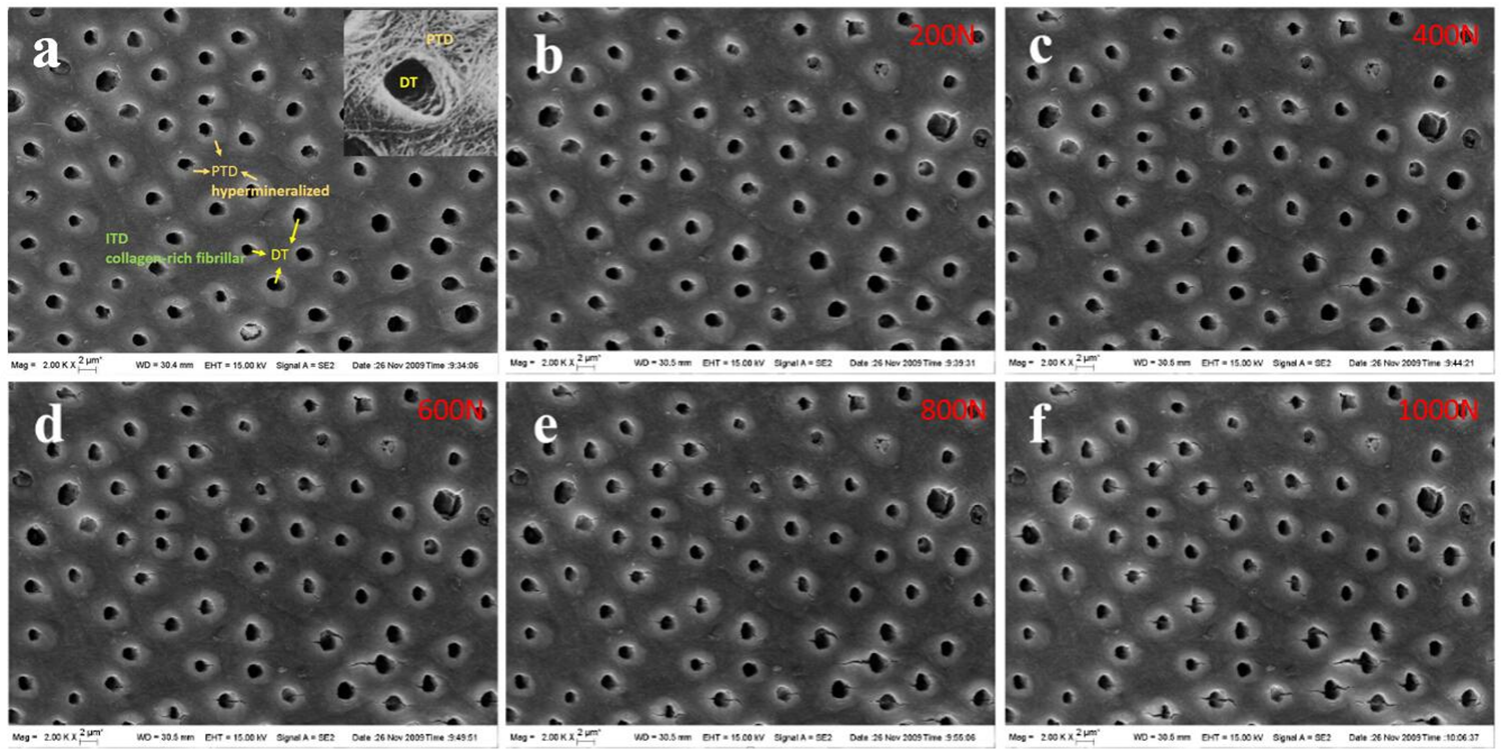
Tubules in dentine as seen in SEM. An image of 72 tubules observed in one of the backscattered SEM images of a polished slice of dentine. The inset shows a magnified tubule which includes a void and a dense peritubular sheath. (a) ~ (f) are the original image under the compression load, e.g., 200 N, 400 N, 600 N, 800 N and 1000 N, respectively.

Dentine samples were carefully placed into the scanning electron microscope chamber (SUPRA55, Germany) after being coated with metal (Denton Vacuum, USA). To obtain a high quality image, the conducting resin was placed lateral to the sample. Compression loading was set at the rate of 0.1 mm / min so that *in-situ* changes in the surface morphology could be observed. Loading direction was perpendicular to the DT direction. With loading held at 200 N, 400 N, 600 N, 800 N, and 1000 N, SEM photographs were recorded in Fig 2. The geometric parameters of the dentine microstructure were then measured from the recorded images at the specified levels of forces using Image-Pro Plus (IPP) software. It is important that the compression stiffness could been calculated by load-displacement curve which obtained during the compression test. It is concluded that the compression stiffness is approximately 15.12 GPa.

### Finite element simulation

Dentine can be modeled as a continuous reinforcement composite. This model includes peritubular dentine, intertubular dentine, and dentinal tubules as shown in Fig 2a. The DT is surrounded by a cylindrical shaped layer, called PTD. The PTD in transverse cross section appears as ring-like structures. It is a hypermineralized tissue, because there is no collagen. The rest of the tissue around the DTs is ITD, a collagen-rich fibrillar network.

Due to the complicated topological structure of dentine and the uncertainty distribution of DTs, we introduce more than one of its components into an analytical model leads to intractable solutions, however methods such as the finite element method (FEM) offer an alternative approach [11–13]. The FEM has been extensively used for evaluating the stress and strain responses of human teeth under various test conditions. In most of these studies, dentine has been modeled as a homogeneous and isotropic material [14]. In mathematical modeling, dentine is generally simplified as a system of geometrical areas containing only PTD, DTs and ITD. However, these simplifications lead to approximate results, as PTD is considered important.

The equivalent stiffness of the dentine is defined to describe the dentine mechanics. When dentine is compressed, internal pressure is increasing in an enclosed cavity. It causes the compression of internal fluids in DTs which accelerates fluid to flow to the dental pulp. Then it stimulates the dental pulp nerve of the teeth root. In the end, the neuronal signaling is increased until it tranmits to the brain. For modeling purpose, dentine stiffness is correlated with flow rate and nerve stimulations, which could then be studied by FEM under the given dentine configuration.

We use the Image-Pro Plus (IPP) software to create the FE model. The IPP software could read and write image data in all the standard image files formats including the scanning electron microscope image. It could trace and count objects manually or automatically. Measure object attributes such as: area, angle, perimeter, diameter, roundness and aspect ratio. Based on IPP feature, we obtain the location, count, and diameter of PTD and DTs using the original SEM image. The IPP software could also calculate the mean diameter of object. As the PTD and DTs are not totally regularity and PTD is not uniformly distributed around DTs, the mean diameters of PTD and DTs are required to analyze the model correctly. After obtaining the coordinate and mean diameter of PTD and DTs, the model is created and used to perform the finite element analysis.

So just one clear image is needed to create the basic FE model. Based on the above results, we modify the base model like decrease or increase the radius of PTD and DTs by manually to create models with different porosities. The compression stiffness in experiment and the equivalent stiffness in finite element analysis (FEA) are the same description about dentine strength.

**Fig 3:**
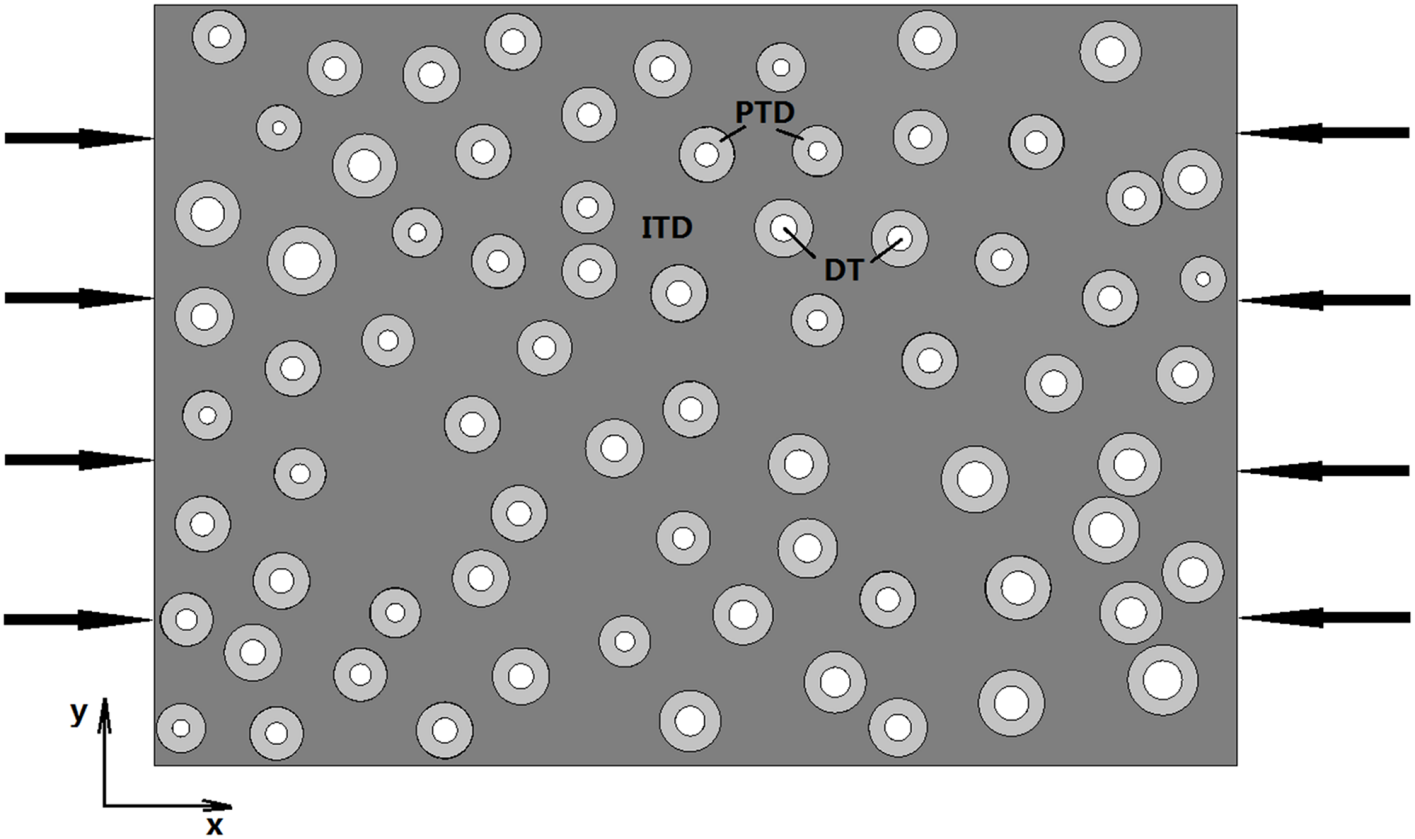
Schematic representation for the compression and boundary conditions of dentine. The model of dentine based on SEM image and the white void presents DT, light grey phase presents PTD, the dark grey phase presents matrix ITD.

ANSYS software is used to analysis. The finite element package is used to calculate the dentine equivalent stiffness. In finite element analysis, compression loading is set at both sides along the *x* axis as shown in Fig 3. Then, the model is discretized using PLANE183 for 2D modeling of a solid structure defined by eight nodes with two degrees of freedom at each node: translations in the nodal *x* and *y* directions. The boundary conditions: the middle points of left and right sides are fixed in *y* direction, and middle points of top and bottom sides are fixed in *x* direction. After solving, the data could obtain in post-processing. The data mainly contains the displacement of the two loading sides. Then the strain is acquired. According to the experiment compression loading and the loading area of sample, the compression pressure is calculated about 100 MPa. So the equivalent stiffness is defined as the compression pressure divided by the strain. The compression stiffness is considered as the verification results to finite element analysis results. The material parameters of dentine model and the comparison results under 1000 N as the following Table 1 shows.

**Table 1:** Comparison of stiffness results between experiment and FEA.

	*E*_*p*_/GPa	*E*_*i*_/GPa	porosity	*m* (*R*_PTD_/*R*_DT_)	stiffness/GPa	relative error
Experiment	26	15	4.38%	2.32	15.12	0.80%
FEA	26	15	4.44%	2.33	15.00	

where the *E*_*p*_ is the modulus of PTD, *E*_*i*_ is the modulus of ITD and the dimensionless parameter *m* is the ratio of *R*_PTD_ to *R*_DT_. The stiffness relative error between the experiment and FEA is about 0.80%. Therefore, the results show that the FE model in our present study is effective. And it provides the basis for the subsequent topological structure study. So in the following study, the different FE models are determined from manually assigned. In other words, we just increase or decrease the radius of PTD and DTs by manually assigned.

## Results

### The effect of PTD on stress and displacement distribution

**Fig 4:**
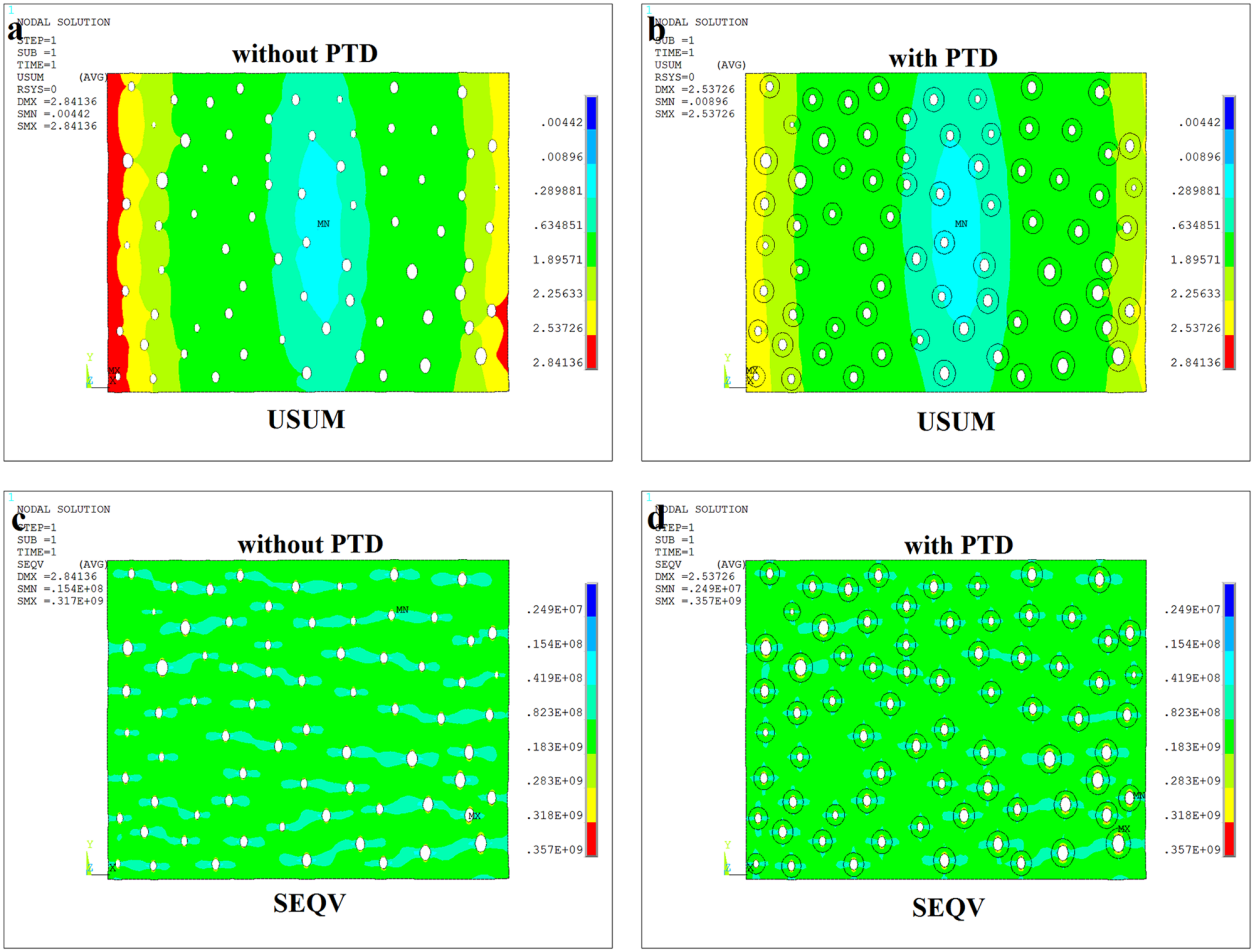
The FEA results of dentine section model. (a) The displacement contour plot of dentine section without PTD; (b) The displacement contour plot of dentine section with PTD; (c) The Mises stress contour plot of dentine section without PTD; (d) The Mises stress contour plot of dentine section with PTD.

In order to study the effect of PTD on stress and deformation distribution in dentine, two different scenarios are modeled: with and without PTD in the structure. The boundary conditions and the distribution of DTs are just the same, the only different from the two kinds of models is the PTD. The FEA results are plotted in Fig 4. Fig 4a and 4c are the results of dentine models without PTD and Fig 4b and 4d are the results of dentine models with PTD. From the FEA results, we found that the existence of the PTD layers have a great effect on both the stress and displacement distribution. As seen in the comparison between the displacement results of Fig 4a vs 4b, the displacement without PTD is larger than that with PTD. This suggests that PTD could decrease dentine displacement and reduce the destruction. As seen in the comparison between the Mises stress distribution results of Fig 4c vs 4d, PTD decreases the interference between the DTs. And in Fig 4c, the interference between DTs is obvious, especially when the distance between DTs is small. It could occur crack initiation and propogation more easily. However, there is less interference in Fig 4d. In Fig 4b and 4d, we also see that there is no significant interference between the neighboring DT and PTD elements most. However, in the bottom right area of Fig 4d, the interference seems obvious. It is basically same with the experiment results in Fig 2c~2f which present the five different loading results. So the comparison results show that the finite element method is reasonable and correctly in this study. We could use this method to proceed the next study.

From the results of Fig 4, one can conclude that the effect of PTD on the porous dentine is to reduce stress and deformation; that is, the dentine microstructure with PTD is the biologically optimized microstructure. Mechanically, PTD is peritubular reinforcement phase. First, PTD prevents the interference between DTs with the reinforcement; then, PTD prevents the generation and propagation of the crack which starts at the edge of the DT; most importantly, PTD increases the strength and stiffness of the whole dentine structure. Therefore, we next analyze the equivalent stiffness in the individual elements and take into account the peritubular reinforcement effect (PRE) of PTD on dentine structure.

### The effect of porosity on equivalent stiffness

In this section, the ratio of *E*_*p*_ to *E*_*i*_, porosity and the ratio of *R*_PTD_ to *R*_DT_ which influence the equivalent stiffness are discussed. Where *E*_*p*_ and *E*_*i*_ are the elastic modulus of PTD and ITD, respectively. And *R*_PTD_ and *R*_DT_ are the radius of PTD and DT. The *δ* is the porosity, which is defined as the percentage of the area of DTs to the entire force area. The thickness of PTD is just calculated by *R*_PTD_ subtract *R*_DT_. The change in equivalent stiffness is observed under restrictions. The numerical simulations and boundary conditions are the same with Fig 3.

It is known that, the greater of porosity, the greater effect on equivalent stiffness [15, 16]. We thus evaluate the change of equivalent stiffness in the certainty distribution of DTs. A reasonable estimate of the equivalent stiffness can be made by considering the porous phase as inclusions distributed in the solid phase randomly. Therefore, the porous material is absolutely characterized by the volume fraction of the void. The presence of voids affects the overall mechanical and physical properties of the dentinal material. In order to calculate the equivalent stiffness of porous material, the material can be treated as a composite medium composed of a homogeneous matrix and a large number of circle inclusions.

The modulus of PTD and ITD are influenced by many factors, and the measured modulus varies in published literature. To better describe the effect of PTD and ITD on equivalent stiffness, *E*_*p*_/*E*_*i*_ is used to compare the equivalent stiffness and obtain the optimized ratio of *R*_PTD_ to *R*_DT_. Kinney et al [17], used an atomic-force microscope indentation technique to determine the Young’s modulus of the peritubular and intertubular dentine and estimated them to be approximately 30 GPa and 15 GPa respectively. Misra et al [18], calculated the Young’s modulus of the peritubular and intertubular dentine as approximately 26~30 GPa and 13~20 GPa respectively. Based on these results, the ratio of *E*_*p*_ to *E*_*i*_ is calculated as 2, 1.73 and 1.

**Fig 5:**
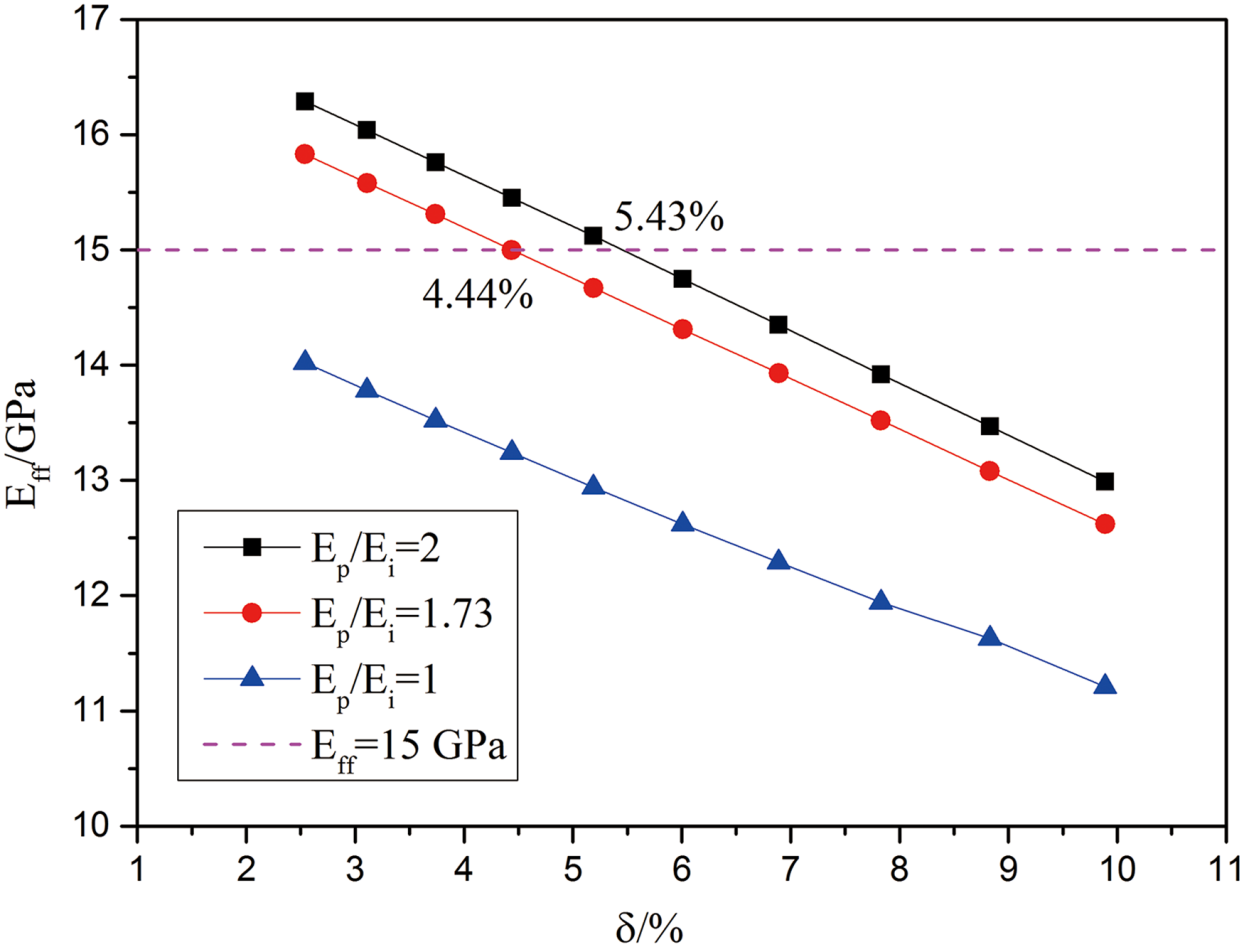
The relationship between *E*_*ff*_, porosity and *E*_*p*_/*E*_*i*_ when the *R*_PTD_ is kept the same.

Further in the discussion of *E*_*ff*_, *δ* and *E*_*p*_/*E*_*i*_ when *R*_PTD_ is kept the same, *E*_*i*_ is defined as 15 GPa presented in the dotted line. When the *R*_PTD_ is kept the same but with increasing porosity, which means the *R*_DT_ increases, the results of *E*_*ff*_ with different *E*_*p*_/*E*_*i*_ are plotted in Fig 5, respectively. If *E*_*p*_/*E*_*i*_ = 1 is used as the reference, we found that *E*_*ff*_ decreases with increasing porosity. When *E*_*p*_/*E*_*i*_ = 2 and *δ* = 5.43%, *E*_*ff*_ would be 15GPa which is also the modulus of the matrix ITD. When *E*_*p*_/*E*_*i*_ = 1.73 and *δ* = 4.44%, *E*_*ff*_ would almost 15GPa. So when *R*_PTD_ is confirmed, with the decreases of *E*_*p*_/*E*_*i*_, the *E*_*ff*_ is decreasing. These will also results in decreased porosity to achieve the strength and stiffness requirement. Thus, in order to get the modulus of the matrix at least, the different *E*_*p*_/*E*_*i*_ corresponds to different porosity. Examining from a different perspective, the presence of porosity which is considered as the pore defects could be compensated by adjusting the thickness or the radius of PTD to ensure preserved stiffness in the porous dentine.

In our experiment model, the *E*_*p*_/*E*_*i*_ is 1.73. Basing on the above discussion, we would expect the porosity to be 4.44% in order to avoid reduction in dentine stiffness. In fact, our calculated porosity based on the SEM image is 4.38%, which is very close to the FEA result. So these results could confirm the reliability of our FEA results.

### The effect of *R*_PTD_/*R*_DT_ on equivalent stiffness

PTD thickness is known to vary between individuals and with age [19]. The lower values for strength may be the result of the comparatively larger size of the average dentine tubule diameter (say 1 μm) [20]. PTD thickness is about 0.8~2.0 μm and with a radius of 1.65~1.7 times than the radius of the DT [4, 21, 22].

In this study, the thickness of PTD is just calculated by *R*_PTD_ subtract *R*_DT_. Then, the question is how much would PTD thickness need to vary to ‘compensate’ the decreasing stiffness which associates with DTs. We introduce the dimensionless parameter *m*, which is defined as the ratio of *R*_PTD_ to *R*_DT_, to describe the relationship between the equivalent stiffness and PTD thickness. The underlying hypothesis is that PTD evolves around DTs to enhance the dentine stiffness.

**Fig 6:**
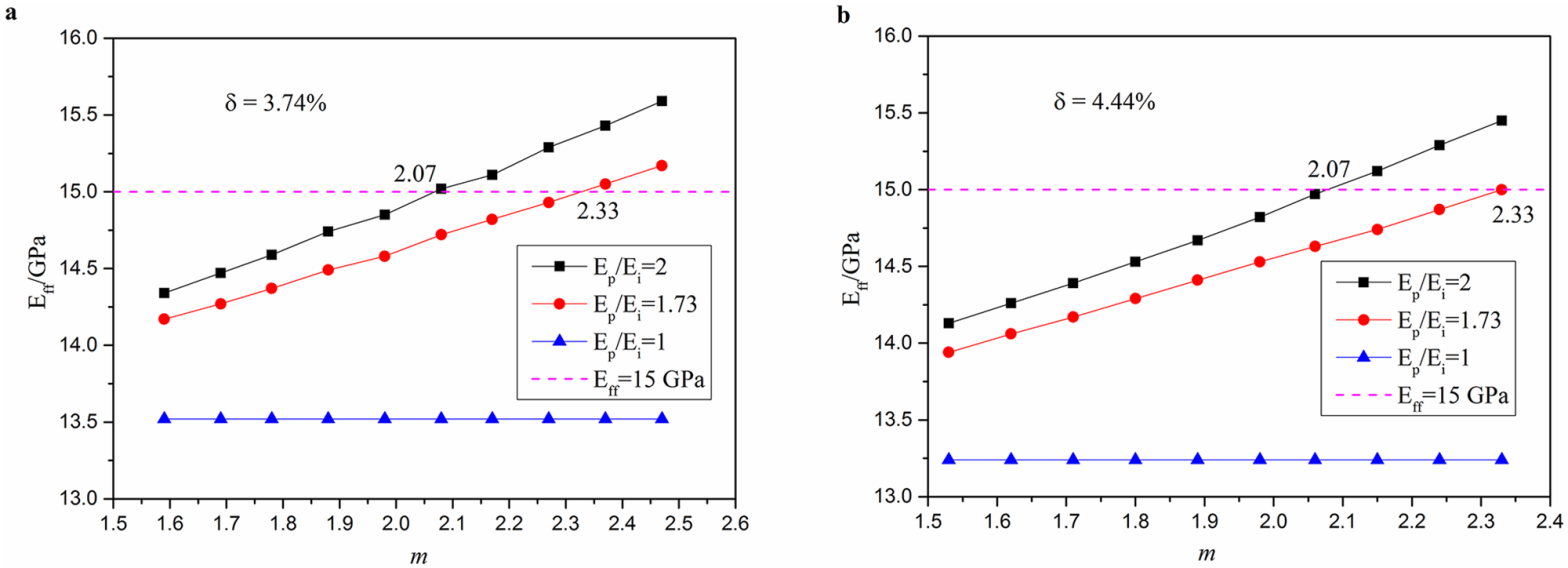
The relationship between equivalent stiffness, *m* and *E*_*p*_/*E*_*i*_ when the porosity is kept at 3.74% and 4.44% respectively.

From the SEM images, we found that the PTD do not overlap with each other. Then the maximum and minimum porosities are obtained. In our experiment, the porosity which we statistics is about 3~6%. In our FE models, we calculate the two different results when the porosities are 3.74% and 4.44%, respectively. The results are shown in Fig 6.

If the porosity is kept at 3.74% while *m* increases, which means the radius of PTD or the thickness of PTD is increasing, the variation trends of *E*_*ff*_ are plotted in Fig 6a with the *E*_*p*_/*E*_*i*_ is 2, 1.73, 1. When the ratio of *E*_*p*_ to *E*_*i*_ is greater than 1, the *E*_*ff*_ increases. When the value of *E*_*p*_/*E*_*i*_ gets closer to 1, the variation trends are not obvious. When *E*_*p*_/*E*_*i*_ = 2 and *m* = 2.07, the *E*_*ff*_ could get 15 GPa which is the modulus of the matrix ITD. When *E*_*p*_/*E*_*i*_ = 1.73 and *m* = 2.33, the *E*_*ff*_ could also get 15 GPa. The same situation happens in Fig 6b. When the porosity is 4.44%, *E*_*p*_/*E*_*i*_ = 2 and *m* = 2.07, the *E*_*ff*_ could get the modulus of the matrix. When *E*_*p*_/*E*_*i*_ = 1.73 and *m* = 2.33, the *E*_*ff*_ could also get the modulus of the matrix 15 GPa.

**Fig 7:**
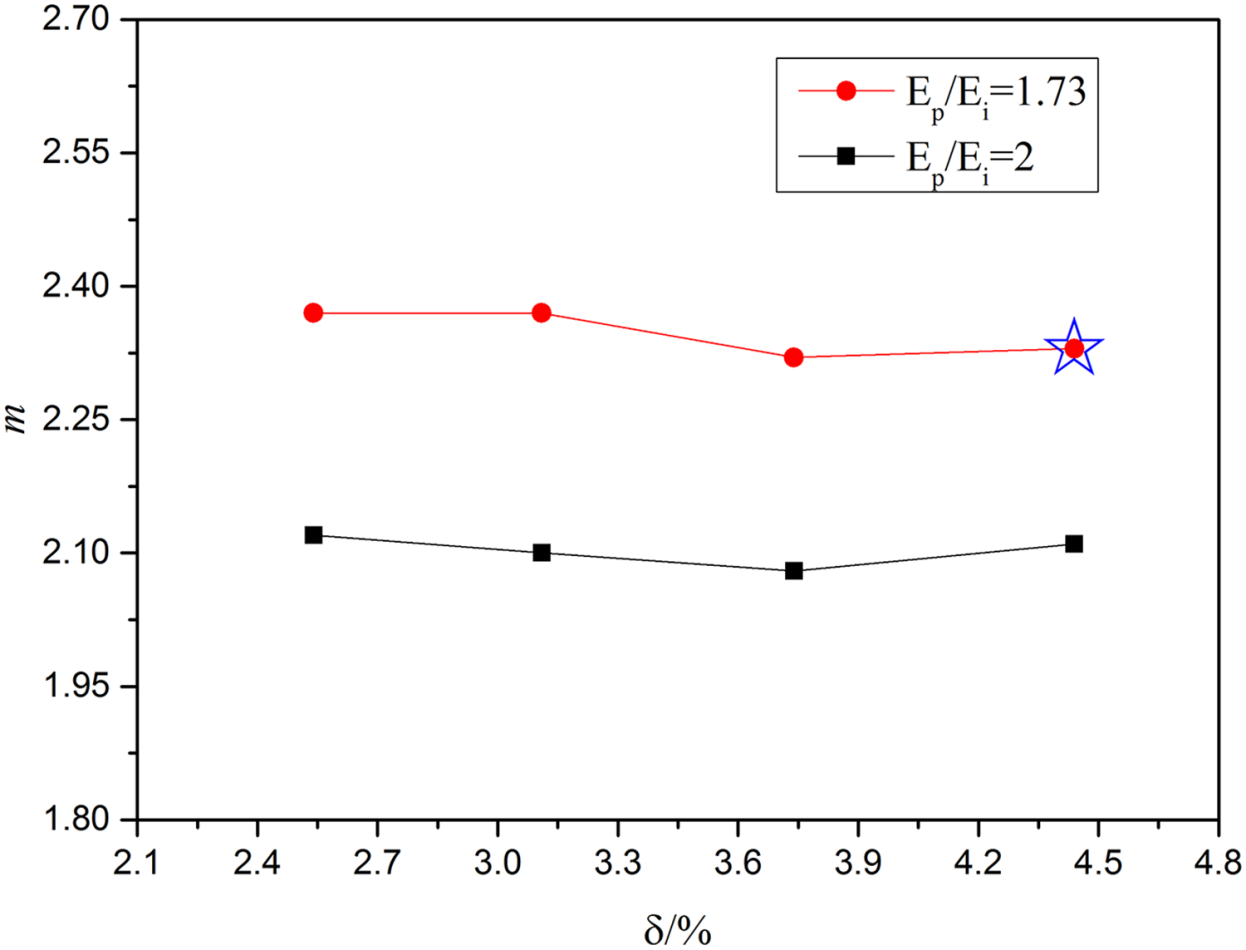
The relationship between porosity, *m* and *E*_*p*_/*E*_*i*_.

To verify the above two results, numerical calculation about *m* is evaluated using different porosities (Fig 7). The matrix ITD modulus is defined as 15 GPa. When the *E*_*p*_/*E*_*i*_ is 1.73 which means the *E*_*p*_ is 26 GPa, the mean *m* is about 2.33 with the porosity of 2.54%, 3.11%, 3.74% and 4.44%. The results illustrate that when *R*_DT_ is determined, when *m* is about 2.33 which could ensure the whole stiffness of porous dentine is not decreased. When the *E*_*p*_/*E*_*i*_ is 2 which means the *E*_*p*_ is 30 GPa, the mean *m* is about 2.07 with the porosity is 2.54%, 3.11%, 3.74% and 4.44%. These results would ensure that dentine stiffness does not decrease. In other words, the greater of *E*_*p*_ with the smaller of PTD thickness could be used to ‘compensate’ to the reduction in stiffness of dentine. In Fig 7, the experimental sample result which we test has been marked using a five-point star. It shows that in SEM image dentine model, the porosity is 4.38% when *m* is 2.32, which is very close to our FEA result (4.44%).

The above results show that once the *E*_*p*_/*E*_*i*_ is determined, regardless of the porosity, the *m* is unchanged. So we could obtain the thickness of PTD basing on the value of *m* to achieve the objective of preventing reduction in stiffness. Therefore, in order to ensure that the stiffness of the composite structure is not lower than the matrix stiffness, once the porosity and *E*_*p*_, *E*_*i*_ or *E*_*p*_/*E*_*i*_ is confirmed, the thickness of PTD is also confirmed. This would avoid the loss of stiffness and ensure the stiffness of the dentine composite structure. It also would explain the biologically optimized microstructure basing on the existence of porous dentine.

## Discussion

There are thousands of dentine tubules extend from the inner pulp space to the outer surface. This structure determines the dentine is a permeable mineralized tissue. Their densities in dentine are about 10,000 to 96,000 mm^-1^ [23]. Based on some results, we found that the dentine tubules and peritubular dentine are both wider at the outer depth than at the inner regions. Some results show that as the different between the thickness of peritubular dentine of bovine dentine and the human dentine, which results the structural properties of bovine dentine are different from the human dentine [24]. This is because the needed bite force and the different function between the bovine dentine and the human dentine. Scanning of selected regions of interest enabled identification of both peritubular and intertubular dentine, it reveals that there is a significant heterogeneity in the mechanical properties in these regions [10]. Scanning based nanoscopic dynamic mechanical analysis shows that the old dentine tubules exhibit a gradient in elastic behavior, with a decrease in elastic modulus from the peritubular dentine to the center of dentine tubules [21]. The mercury intrusion porosimetry technique is used to measure the porosity and quantify the porous distribution [25]. In order to determine the mechanical properties, a micro-mechanics cell model is used to describe the dentine structure [26]. And the Martini [16] found that the maximum stress could move from the peritubular dentine to hybrid layer. The effect of the orientation of dentine tubules on mechanical properties and deformation is studied by stress-strain profile. Results show that the elastic modulus and the yielding stress and strain at various orientation angles [27]. Within physiological limits, the strain-stress response of dentine has outstanding features which are similar to cellular solids [28, 29]. With isotropic elastic modulus, the loading direction orthogonal to the compressive stresses and strains concentrates. These results show that around the dentine tubules, dentine experiences circumferential deformations during the compressive loading.

In dentine, the mineral constituents contribute the stiffness of dentine, and the high mineralization with high elastic modulus and static strength but low strain energy and stiffness [30]. The organic and collagen fraction provide the toughness and stiffness. The optimized functionality of the overall tooth structure is mainly influenced by the gradient mineralization. And the gradient mineralization is mainly affected by the collagen mineralization degree which changes constantly with location in dentine [31]. During the ‘service period’ of dentine, its mainly sustain the compression loading and cycling loading. In cycling loading, the main failure manifestation is the microcrack. One of the major damage mechanisms during cycling loading in tubule system is the initiation and propagation of microcracks along the tubule process. Once exceeding the critical threshold, the system becoming unstable. It has been demonstrated that the tubular and peritubular system restrain failure through crackbridging and frictional pullout [32]. The dentine submitts to cycling loading could compensate for the initiation of macrocracks by nucleating minerals at micro and nano-scale damage zones. These bridge-like structures are also observed in other locations, and have contributed to enhance the long-term behavior of composites under cyclic loading [32].

Although there are many studies examining PTD and DTs, few examine PTD thickness and porosity in relation to the strength and stiffness of the composite dentine structure. It is difficult however to understand the function for the PTD, although its porosity does provide means for the transport of ions and other components between the odontoblast processes and the dentine matrix.

In classical mechanics, DTs are considered as defects or voids in dentine which result in the loss of equivalent stiffness of dentine. In order to improve the dentine stiffness, the PTD plays an important role in enhancing the equivalent stiffness of dentine. Mechanically, the modulus of PTD is much larger than ITD; thus, PTD is considered as the peritubular reinforcement phase. On the other hand, the biological functional of the dentine structure is not only to make sure that the DTs transport nutrients normally, but also to sustain the strength of the composite dentine structure for daily function, such as chewing. In the current manuscript, we perform numerical modeling for the optimal microstructure based on a biologicaly porous dentine, which mainly aims to explain the optimized development of porous dentine with the PRE on dentine biomedical porous materials.

Our approach involves an analysis of the effects of PTD thickness and radius on dentine stiffness. This is done by comparing the models with and without the PTD based on the IPP software, and vertifying the FEA results with experiment. The measured radius and location of the PTD and DTs corresponded well with our FEM estimate. So we believe that this approach has wide potential application.

Finite element analysis is an analytical method for better to study the dentine microstructure properties. As we discuss different models in this study. However, we could not test so many dentine specimens to create the models. So we regulate the ratio of *R*_PTD_ and *R*_DT_ to accord with the dentine models as soon as possible meanwhile ensure the continuity of models and results. And the FEA could calculate different dentine models correctly. First, we just consider the peritubular phase effect on equivalent stiffness of dentine model by finite element analysis. Its try to use the simple model to solve the complex problem. So the 2D FE model is totally could solve these dentine models. Besides, the characteristic length of microstructure is negligible compared to the thickness of the dentine tubules. Therefore, the 2D plane strain is assumed in the FEA which can be used to study the stiffness property. In other words, the 2D plane strain FE model is suitable to represent the approximate a 3D dentine structure.

The manually assigned parameters mainly contain the porosity and *m*. In other words, the different stiffness for corresponding models are calculated just by changing the radius of PTD and DT of Fig 3 model. In porosity discussion, increasing porosity means increase the radius of DT while the radius of PTD remains the same. So in this part, it mainly adjusts the radius of DT. The results show that with the increasing of the porosity, the equivalent stiffness is decreasing. In certain *E*_*p*_/*E*_*i*_, the compensate porosity is given which could ensure that the whole equivalent stiffness wouldn’t decrease. The thickness of PTD is proportional to the equivalent stiffness. It is shown in Fig 5. In *m* (ratio of *R*_PTD_ and *R*_DT_) discussion, increasing *m* means increasing the radius of PTD while the radius of DT or the porosity remains the same. So in this parts’ discussion, it mainly adjusts the radius of PTD. In Fig 6, two kinds of porosity are discussed. And in certain *E*_*p*_/*E*_*i*_, the compensate *m* is given which could ensure that the whole equivalent stiffness wouldn’t decrease. In the end, we take multi-factor, porosity and *m*, into comprehensively considering to discuss the relationship among the porosity, *m* and the whole equivalent stiffness of dentine. And the porosity and *m* are two factors which all have relation with peritubular thickness. The results are shown in Fig 7.

Our results demonstrate that the PTD appear thicker around the smaller DTs. This is in accord with other reports of stiffening and strengthening of the dentine microstructure [33] which can be explained by increased PTD thickness. Further analysis is still needed to fully characterize the distribution and thickness of the PTD in dentine. As shown in Figs 6 and 7, we found that the PTD thickness and porosity are the main factors influencing equivalent stiffness. The smaller the radius of DT, the thicker the PTD. With increased porosity, the PTD also increased. But there is the limit to the increasing boundary, which is that there is no overlap between PTDs. With different *E*_*p*_/*E*_*i*_ and porosity, the *m* (*R*_PTD_/*R*_DT_) is different. So the relation between the radius of PTD and DT can be calculated. In our 72 DTs’ experimental configuration, the modulus of PTD and ITD is 26 GPa and 15 GPa, respectively. So *E*_*p*_/*E*_*i*_ is about 1.73 and the porosity is about 4.38%. From the above results like Fig 6b, the *m* is about 2.33 which means the *R*_PTD_/*R*_DT_ is about 2.33. We found that the mean value of *R*_PTD_/*R*_DT_ in our dentinal configuration is about 2.32 which corresponds well with our findings in Fig 7 and those in the literature [33].

Mechanical properties of dentine with age have largely been attributed to the increase in mineralization due to filling of the dentine tubules. Human teeth undergoes changes with increasing age, there are difference in mechanical properties, such as an increase in elastic modulus and hardness, a decrease in strength. Besides, the increase in mineralization of the dentine tubules which could cause some trouble in modeling and the statistics porosities of dentine. For example, the dim boundary between PTD and DT. Therefore, the age has a great effect on stiffness property and microstructure of dentine. We have noticed these characteristics. However, we mainly pay attention to the equivalent stiffness of young people dentine in order to decrease or eliminate the affection of other factors. The present method is suitable to study the mechanical properties of teeth in a large range of age.

There are variations in composition in restorative materials between anterior and posterior teeth. However, aesthetics factor should be considered in restorative materials of anterior teeth firstly. It mainly includes such as color, gloss and permeability. Whereas the function of posterior teeth is chewing. So the restorative materials of posterior teeth should consider the mechanical property (strength and stiffness) which could bear the bite force. Therefore, the third molars are chose to test. And the SEM image of the third molars specimen is regarded as the basic FE model.

Porous material are widely used in clinical and engineering structures although the porosity results in decreasing the material strength. In engineering, material stiffness of porous material can be reinforced by adding the reinforcement materials around the porosity. Our present findings suggest that with the dentine structures evolving, they came up with this same biological solution. Thus, these findings are also broadly applicable to other biomaterials and to the engineering of porous materials.

## Conclusions

In this study, we develop a mechanical model to simulate peritubular reinforcement effect of porous dentine tubules. We report that increasing PTD thickness could result in improving the composite dentine stiffness. The biological significance is that the existence of the PTD reduces the stress concentration around DTs and the destruction of the DTs. Particually, the PTD strengthens the stiffness of the composite dentinal structure. And it is the “biological solution” which is utilized by this microstructure to compensate the stiffness of porous dentine. These results are applicable to other porous materials in engineering which could use this method to strengthen the structure by adding the reinforcement materials around the porosities.
